# Bacteroidetes use thousands of enzyme combinations to break down glycans

**DOI:** 10.1038/s41467-019-10068-5

**Published:** 2019-05-03

**Authors:** Pascal Lapébie, Vincent Lombard, Elodie Drula, Nicolas Terrapon, Bernard Henrissat

**Affiliations:** 1 Architecture et Fonction des Macromolécules Biologiques (AFMB), Centre National de la Recherche Scientifique (CNRS, UMR7257), Institut National Agronomique (INRA, USC 1408) and Aix-Marseille Université (AMU), 13288 Marseille cedex 9, Marseille, France; 2 Department of Biological Sciences, King Abdulaziz University, Jeddah, Saudi Arabia

**Keywords:** Carbohydrates, Glycobiology, Genome informatics, Bacterial genomics

## Abstract

Unlike proteins, glycan chains are not directly encoded by DNA, but by the specificity of the enzymes that assemble them. Theoretical calculations have proposed an astronomical number of possible isomers (> 10^12^ hexasaccharides) but the actual diversity of glycan structures in nature is not known. Bacteria of the Bacteroidetes phylum are considered primary degraders of polysaccharides and they are found in all ecosystems investigated. In Bacteroidetes genomes, carbohydrate-degrading enzymes (CAZymes) are arranged in gene clusters termed polysaccharide utilization loci (PULs). The depolymerization of a given complex glycan by Bacteroidetes PULs requires bespoke enzymes; conversely, the enzyme composition in PULs can provide information on the structure of the targeted glycans. Here we group the 13,537 PULs encoded by 964 Bacteroidetes genomes according to their CAZyme composition. We find that collectively Bacteroidetes have elaborated a few thousand enzyme combinations for glycan breakdown, suggesting a global estimate of diversity of glycan structures much smaller than the theoretical one.

## Abstract (web summary)

Bacteroidetes genomes contain polysaccharide utilization loci (PULs), each of which encodes enzymes for the breakdown of one particular glycan. By analyzing the enzyme composition of 13,537 PULs, the authors suggest that the natural glycan diversity is orders of magnitude lower than previously proposed.

## Introduction

**Fig. 1:**
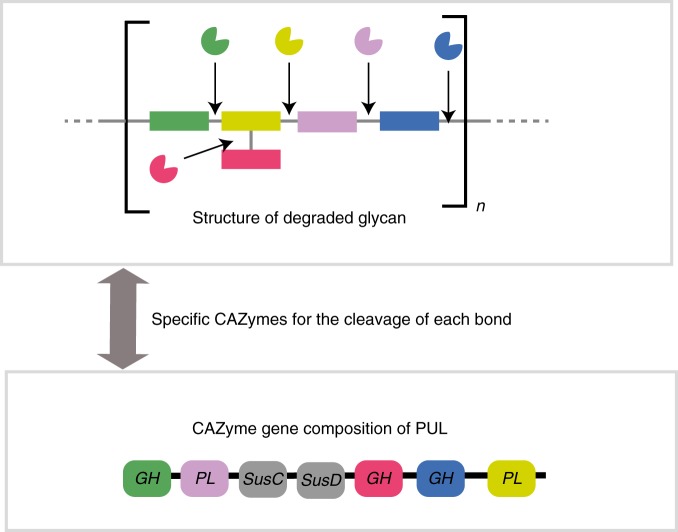
Schematic view of the approach taken to estimate the number CAZyme combinations in PULs. The depolymerization of a given complex glycan by Bacteroidetes PULs requires bespoke enzymes secreted in the perisplasm and the extracellular milieu. Conversely, the enzyme composition of PULs provides information on the structure of the targeted glycan. The enumeration of PULs according to their composition encoded CAZymes gives an estimate of the diversity of glycans degraded by Bacteroidetes

**Fig. 2:**
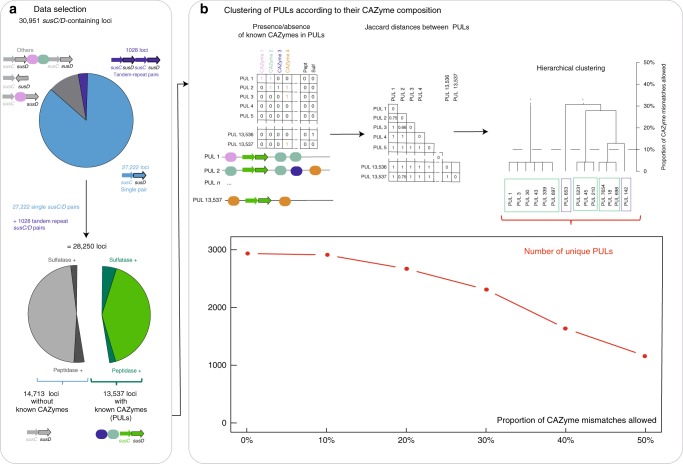
The PUL analysis pipeline. **a** Selection and sorting of data from PULDB. **b** Clustering of PULs according to their CAZyme composition. The distance between each pairs of PULs has been calculated according to the composition in enzyme (sub)families. Hierarchical clustering with different distance thresholds (from 0 to 50% mismatch in CAZyme composition) yields a number of unique PULs between ~1200 and ~2900

Intrinsically associated with life, especially since the emergence of photosynthesis, glycans are the main form of energy storage by (photo- or chemo-synthetic) autotrophic organisms, which produce the dominant biomass on the planet^1^. In addition, glycans have structural roles for cells (i.e., extracellular matrix) as well as for whole organisms (i.e., exoskeleton) and important roles as signaling molecules. Whilst the major polysaccharides are well known (cellulose, chitin…), the natural diversity of glycan structures remains uncharted. In contrast to proteins, the primary structure of glycans is often branched due to the multiple hydroxyl groups on each carbohydrate monomer. Laine has calculated that there could be over 10^12^ possible isomers for a hexasaccharide^2^. The enzymes that break down glycans (CAZymes; Carbohydrate-Active Enzymes) often display exquisite specificity that distinguishes the carbohydrate moiety and type of glycosidic bond. Thus while just a few highly conserved families of proteases can virtually break down all proteins^3^, the degradation of complex glycans has resulted in the evolution of numerous and highly diverse families of CAZymes. Reciprocally, the specificity of degradative CAZymes can provide information on the structure of the degraded glycans^4^ (Fig. 1). Among the heterotrophic bacteria, the phylum Bacteroidetes, which comprises seven classes including Bacteroidia, Cytophagia and Flavobacteriia^5^, is present in all ecosystems^6^ from deep oceans^7^ to desert sand^8^. In Bacteroidetes glycan-degrading systems, secreted CAZymes partially depolymerize the polysaccharide to large oligomers that are imported into the periplasm by transporters encoded by a *susC/D* gene pair, and subsequently degraded in the periplasm by other sugar-cleaving enzymes, away from competing organisms^9^. All genes encoding enzymes, transporter and regulators that target a specific glycan are located on the same portion of the genome; these regions are termed polysaccharide utilization loci (PULs)^9–11^. Functional characterization of the enzymes encoded by PULs has shown that each PUL appears dedicated to the breakdown of a particular glycan structure^4,12–15^. Here we analyze the wealth of genomic data available for Bacteroidetes from many environments to estimate how many enzyme combinations have been assembled by Bacteroidetes to break down glycans (Fig. 2). We propose that this ensemble of combinations represents a proxy for glycan diversity based on real experimental biological and biochemical data.

## Results and Discussion

### Data selection

The Polysaccharide Utilization Loci database (PULDB; www.cazy.org/PULDB) lists both predicted loci and those that have been identified experimentally^10^. The order of genes encoding CAZymes in PULs does not seem to be important^14^. In PULDB, PUL predictions are entirely automated and are based on the identification of *susC-susD* homologs followed by the examination of the occurrence of degradative CAZymes around each *susC/D* pair within a fixed intergenic distance empirically derived from the examination of two *Bacteroides* species that have been subjected to extensive transcriptome analysis in response to various glycans^10^. The list of species in PULDB has been recently expanded^16^ to 964 species isolated from various environments (marine, soil, digestive microbiota) collected in various areas of the globe. To ensure that the PUL prediction algorithm also functioned correctly with more distantly related species, we checked that it was able to retrieve at least 80% of the PULs reported for *Flavobacterium johnsoniae*^17^ a species distant from the two *Bacteroides* that were used to calibrate the PUL predictor (Supplementary Table 1). In order to examine the potential impact of intergenic distances on PUL predictions in the classes of Bacteroidetes, we performed a multidimensional analysis of genomic characteristics (taxonomic class, number of PULs, number of ORFs in PULs, CAZyme content within and outside PULs, intergenic distances within and outside PULs) in the 964 genomes (Supplementary Note 1), but intergenic distances did not explain the inter-class variability.

We first sorted the 30,951 *susC/D-*containing loci (this term designates any group of genes around a *susC*-*susD* gene pair, with or without CAZymes) listed in PULDB, according to their encoded protein category content (Fig. 2a; Supplementary Data 1). 3,317 such loci were rejected because they did not conform canonical PUL composition (without adjacent *susC* and *susD* genes). 1028 loci exhibited an unusual tandem repeat organization of their *susC/D* genes and were kept for further analysis (*vide infra*). Approximately half of the loci containing a *susC/D* gene pair did not harbor any known CAZyme gene (*vide infra*) while the other half (13,537) encode for Carbohydrate Esterases (CE), Glycoside Hydrolases (GH) and Polysaccharide Lyases (PL) currently classified in families of the Carbohydrate-Active Enzymes (CAZy) database^18–20^ (www.cazy.org) and therefore represent PULs. In order to estimate the diversity of the glycans targeted by this set of 13,537 PULs, we compared their CAZyme composition harnessing the substrate specificity found in CAZyme families. The large multifunctional families GH5, GH13, GH30 and GH43 were divided into multiple subfamilies, as the latter have shown much improved correlation with substrate specificity^18,21–24^. In addition to CAZymes, we added two additional broad categories: sulfatases^25^ and peptidases^8^ as these two activities have been found in several PULs, and are biologically relevant since glycans can be sulfated^25,26^ and/or attached to proteins^27^. The complete list of families and subfamilies that have been used for this work is given in Supplementary Data 2.

### Analysis of PUL composition

For the analysis of PUL composition, we chose to compile the presence/absence of glycan-degrading families and not the number of genes (*vide infra*). A presence/absence matrix was generated (Fig. 2b) where each row represents a PUL and each column a CE, GH or PL family (or subfamily), supplemented by sulfatases and peptidases. Out of 13,537 PULs, 90% belong to 1192 groups of at least two PULs of identical enzyme composition while only 10% (1760) have a composition that was encountered only once (singletons; Supplementary Data 3). Thus the number of unique PUL compositions (i.e. groups plus singletons) represents a total of 2952 unique PULs. The median number of different CAZyme (sub)families per PUL is 3.5 + /−0.000028 (95% confidence interval, Wilcoxon test, Supplementary Fig. 1).

The number of these unique PULs is likely to be an overestimate of the diversity of targeted glycans for several reasons. First, different CAZyme families can be isofunctional; for instance, families GH2 and GH147 both contain β-galactosidases but PULs where GH2 is replaced by GH147 are counted as different. Second, a number of genomes are not closed, and in some cases a PUL may be incomplete due to unassembled scaffolds, again resulting in an artificially different composition. Finally, examination of the literature shows that sometimes more than one PUL contributes to the degradation of a given glycan such as fungal α-mannan whose deconstruction relies on three loci^15^.

To estimate the potential reduction of the number of functionally distinct PULs resulting from the above considerations, we examined the effect of allowing a growing number of mismatches in the unique PULs (Fig. 2). From the presence/absence matrix of CE, GH or PL family, we then calculated Jaccard distances^28^ which represent the dissimilarity in CAZyme composition between each pair of PULs. These distances were then used to cluster the set of 2952 unique PULs into groups with a growing proportion of mismatches.

The number of calculated unique PULs as a function of the proportion of allowed mismatches is presented in Fig. 2. For 10% mismatch we observe only a tiny reduction of the number of unique PULs while 20 and 50% mismatch reduce the number of unique PULs to about 3000 and 1200, respectively. Thus we estimate that a few thousand enzyme combinations are necessary for the breakdown of glycans by Bacteroidetes, a number far greater than the number of peptidases required to degrade proteins. The analysis of the 13,058 PULs that we have identified in 964 genomes shows that a few PULs are encountered very frequently (1% were encountered more than 130 times) while 75% of the PULs were found 8 times or less. The complete list of PUL groups is presented in Supplementary Data 4. The abundant PULs target polysaccharides ubiquitous in various environments such as chitin and glycogen/starch but also of less known polysaccharides such as β−1,2-glucans, broken down by GH144 β−1,2-glucanases^29^ (Supplementary Data 3–4). Remarkably, marine organisms have GH144-containing PULs but with the addition of a sulfatase gene, suggesting that a ubiquitous yet unknown marine polysaccharide exists, identical to the terrestrial one in terms of carbohydrates, but decorated with sulfate. Indeed, sulfation of glycans is common in the marine environment^25^, while acetylation is a dominant feature of plant terrestrial polysaccharides^30^.

Enzyme families that target peptidoglycan such as GH23, GH24, GH25 and GH73 are statistically strongly underrepresented in PULs (Supplementary Data 5). This may be due to the fact that these enzymes are often involved in the remodeling of cell wall peptidoglycans during bacterial division^31^ rather than in the utilization of peptidoglycan as a nutritional resource. The starch/glycogen-degrading families GH77 and GH13 (especially subfamilies GH13_8, GH13_9 and GH13_39^24^) were found to be abundant outside PULs and also less frequent in PULs than expected by a random distribution (Supplementary Data 5). This is probably due to the fact that glycogen is also the cytoplasmic carbon storage polysaccharide in many bacteria and its breakdown within the cell does not require PULs. Yet the peptidoglycan- and starch/glycogen-degrading enzymes are found in several predicted PULs, suggesting that their target polysaccharides do not escape degradation by the PUL system.

Among the 13,537 PULs in our data set, 5047 (~40%) have multiple genes coding for the same CAZyme family coinciding with two different scenarios: (i) the different copies may have different specificities and in this case the number of copies should be taken into account to estimate PUL diversity and (ii) the same glycosidic bond may be cleaved in the extracellular medium (to generate long oligosaccharides) and in the periplasm (to achieve complete degradation) by enzymes from the same family. We have thus computed the number of unique PULs taking into account the number of copies of the same family (Supplementary Data 6). Overall, we found that the number of unique PULs increased by ~25% (from 2952 to 3654) but stayed in the same order of magnitude.

In all, 4357 PULs in our data set contain only one family (single family PULs). Of these, 3600 (82%) have only one copy of the gene, 652 (15%) have two copies and only 2% have more than 2 copies. The number of PULs that encode only one CAZyme conflicts with the PUL paradigm, which divides the degradation of a glycan into an extracellular initiation step and one or several intraperiplasmic steps. Two explanations come to mind to explain this apparent discrepancy: (i) not all CAZyme families are known and one cannot exclude that a new family will appear in the vicinity of a single CAZyme gene (ii) a single family PUL may collaborate with other PULs in the degradation for the degradation of glycans. It has already been shown in several cases that the breakdown of a single complex glycan can require several PULs^4,15^. Thus single family PULs may offer flexibility and adaptability to the bacteria to digest complex substrates that display compositional variations. We also noticed that certain families appear significantly more duplicated than others in single-family PULs (Supplementary Data 7). This is especially the case of family GH32 (targeting fructose polymers) and to a lesser extent GH89 and GH33. We also note that all PULs containing only families GH10 and GH55 have two copies of the gene.

### Tandem repeats of *susC/D*-like gene pairs

In the PUL paradigm, the genes encoding glycan-cleaving enzymes are placed around a *susC/D* gene pair that orchestrates the synthesis of a SusC-SusD protein complex whose role is to bind and transport oligosaccharides into the periplasm. Transcriptomic data has shown the presence of a few PULs containing two *susC/D* gene pairs in tandem^32–36^. We found that 1028 of the 30,951 predicted loci (approx. 3%) in our 964 genome data set have directly adjacent pairs of *susC/D* genes, always on the same DNA strand, which we define as *trsusCD* (tandem repeat *susC/D*) (Fig. 2). These atypical PULs, which are proportionally more abundant in the *Bacteroides* genus than in other genera of the Bacteroidetes phylum (Chi^2^ *p*-value < 0.001, Supplementary Table 2), include 1.5 times more protein-encoding genes than typical single *susC/D* PULs (Supplementary Fig. 2) suggesting that they may represent fusions of PULs.

**Fig. 3:**
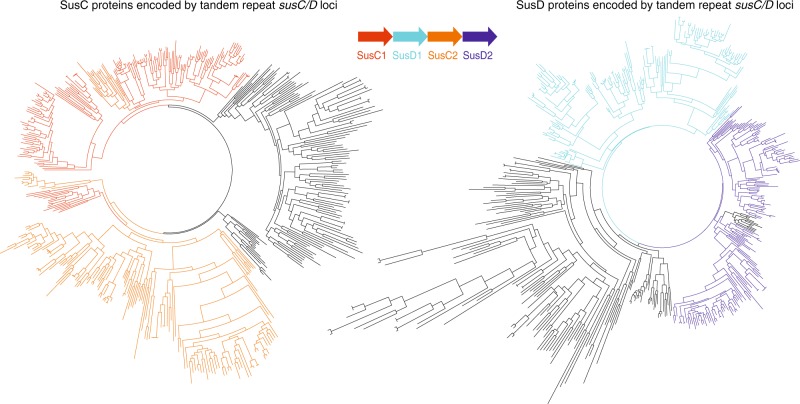
Phylogenetic trees of SusC and SusD proteins encoded by tandem-repeat *susC/D* loci. SusC and SusD form color-coded congruent clades in the two phylogenetic trees. Each member of each clade has same genomic position in the repeat, revealing a strict synteny within each *trsusC/D* groups

In order to examine whether these *trsusCD*-containing PULs are the result of PUL fusion or have a functional significance, we carried out a phylogenetic analysis of SusC and SusD proteins and we distinguished their relative position in the tandem-repeat (SusC1-SusD1-SusC2-SusD2) (Fig. 3). In the reconstructed phylogenetic trees, the amino acid sequences of the SusC and SusD proteins encoded by *trsusCD*s form clades, indicative of distinct groups of SusC-SusD pairs and that the distinct groups correlate with their relative position on the genome (Fig. 3). This strictly conserved synteny argues against a simple fusion of two PULs being the origin of *trsusCD*s, and suggests that extant groups of *trsusCD*s have been under positive selection pressure since they arose. SusC/D protein pairs have been shown to form homodimer complexes^37^. In the case of *trsusCD* PULs, it is conceivable that the two distinct SusC proteins not only form separate homodimers but may also form heterodimers.

The synteny of the *trsusCD*s contrasts with the idea that the order of the CAZyme genes in PULs is entirely unimportant^14^. The availability of a large number of PULs in our sample allowed us to quantify the conservation of CAZyme gene order (synteny) between PULs of identical CAZyme composition (Supplementary Fig. 3). As expected, CAZyme gene synteny decreases with taxonomical distance. Some PULs, however, exhibit unexpected strong synteny across more than four taxonomical classes within the Bacteroidetes (Supplementary Fig. 3), highlighting unexpected selective pressure on CAZyme gene order. For example, PULs with genes encoding CAZymes from families GH30_1 and GH30_3 or PULs harboring GH99 and GH97-encoding genes, show high synteny across taxonomic distance (four examples are presented in Supplementary Data 8). The significance of this synteny is unclear.

Around half (52%) of the 28,250 gene loci containing *susC/D* gene pairs (loci with a single *SusC/D*-gene pair and *trSusCD*s) do not contain any CAZyme-encoding gene (Fig. 2). Among these loci lacking CAZymes, 11% contain predicted sulfatases and peptidases, reported to be often associated with CAZymes in degrading complex carbohydrates^16,25^. This suggests that genes encoding unknown CAZymes, which are not in current CAZy families, may await discovery and characterization. However, another possible explanation for the unexpected proportion of *susC/D* loci without any CAZymes, is that they may be co-transcribed with remote CAZyme genes in the genome. Thus, although not physically linked into PULs, the enzymes and SusC/D protein pairs may still orchestrate the degradation of specific glycans. However, the large number of loci without CAZyme genes also suggests that some loci may target biomolecules other than polysaccharides.

### Loci possibly targeting other biomolecules

We found that among the 14,713 *susC/D* loci without known CAZymes, sulfatases or peptidases (Fig. 2), 576 contain genes encoding proteins annotated as phosphodiesterases (PF00149, PF01663) in the Pfam domain database (Supplementary Data 9). Phosphodiester linkages are common in nucleic acids, suggesting that the latter may represent a target for some loci. Bacteroidetes are naturally competent gram-negative bacteria^38^ and they are necessarily able to import polynucleotides that likely also represent a potential nutrient source. Other macromolecules may also interact with SusC/D transporters. Indeed, the first crystal structure of a SusC/D complex revealed a bound peptide, suggesting a role in peptide rather than oligosaccharide transport^37^.

### CAZyme gene clusters without *susC/D*-like gene pairs

Literature has reported the occurrence of clusters of contiguous CAZyme-encoding genes without a *susC/D*-like gene pair^12^. Such loci may either represent variants of actual PULs or artefactual fragments. In order to examine if these loci correspond to an underestimated diversity of CAZyme compositions, we examined the 2533 GH/PL-encoding loci with no *susC/D*-like genes listed in PULDB^16^ for their potential contribution to diversity. After addition of these *susC/D*-less gene loci to our data set, we examined their presence in the different CAZyme composition clusters (Supplementary Data 10). We found 395 enzyme combinations not already included in any CAZyme composition found in PULs, thus increasing only modestly the diversity estimate. This suggests that most of these loci are fragmentary versions of PULs. This may be due to poor PUL prediction, but it also may correspond to a biological trait in Bacteroidetes that have a tendency to have CAZyme-encoding genes outside their PULs (Supplementary Note 1).

### A rough estimate of glycan diversity

**Fig. 4:**
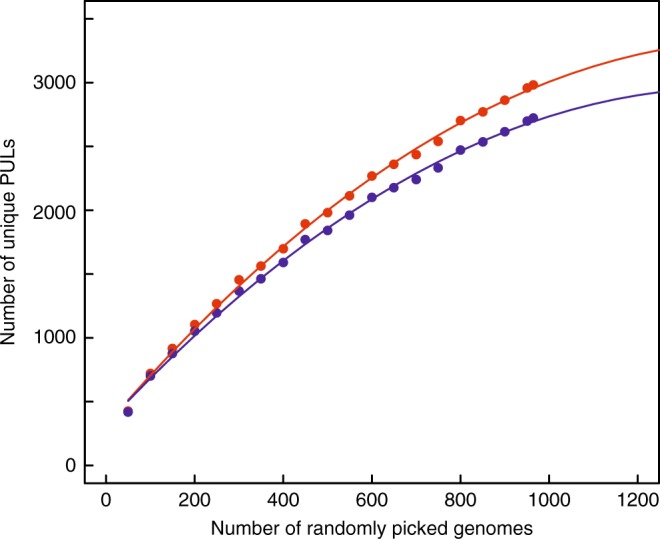
Number of unique PULs according to the number of genomes analyzed. The number of PULs was calculated by randomly resampling an increasing number of genomes from our data set (x-axis). The resampling was performed ten times; the median value is represented on the *y*-axis. A second order polynomial regression gives the trend of two sets of values corresponding to 0 (red) and 20% (blue) mismatch used during PUL clustering

Our estimate of the number of enzyme combinations necessary to break down the diversity of glycans is based on the particular carbohydrate utilization system developed by Bacteroidetes, which differs from other paradigms like cellulosomes^39,40^ or the secretion of free CAZymes^41^. It is possible that some glycans escape the capabilities of PULs. For example, no PUL for the digestion of crystalline cellulose has been reported in the literature, suggesting that crystalline cellulose may evade degradation by dedicated PULs. However, we noticed the presence of PULs containing genes encoding GH5_2 and GH9 cellulases in the *Algoriphagus* genus, suggesting that a β−1,4-linked glucan could be targeted by PUL systems. Although there is burgeoning evidence that gut Bacteroidetes can also degrade bacterial exopolysaccharides^42^, one cannot rule out that some bacterial polysaccharides escape PUL degradation. It is also likely that some of the PULs that we have analyzed target glycans other than those known, or that some CAZyme families that have not been identified so far hide an additional diversity of glycans. Indeed, in the last 12 months the number of known GH families present in PULDB has increased by eight (families GH146–151 and PL27–28). The number of enzyme combinations necessary to cleave glycans will necessarily increase in the future, with the growing number of genomes and of known CAZyme families. In order to distinguish the impact of these two variables on the number of unique PULs, we removed from the current data set the last eight families described during the last year as well as the genomes incorporated during the same period (Supplementary Fig. 4). In one year, the number of genomes appears to be mainly responsible for the description of new PULs (15–20% increase depending on the number of mismatches allowed) while the new GH/PL families contributed just a few percent. To derive a trend for the future, we have thus simulated the number of unique PULs as a function of the number of genomes randomly picked in our data set (values and confidence intervals in Supplementary Data 11). Figure 4 shows that, as the number of new genomes increases, the number of new unique PULs diminishes. Extrapolation of the trend suggests that new unique PULs will appear in the future, but that their number will probably remain in the range of a few thousand.

In summary, our cross-genome study of PULs suggests that the breakdown of natural glycans requires several thousand enzyme combinations. This estimate suggests that the actual diversity of glycans is probably a tiny fraction of the astronomical number based on theoretical combinations of monosaccharides and possible glycosidic bonds^7^. The much lower number of real glycan structures compared to the calculated structures likely reflects the constraints inherent to biological systems at different scales, from steric hindrance, availability of precursors, physiological or ecological constraints^43–45^. In the future, with progress in CAZyme functional prediction, examination of PUL profiles may allow to predict the presence of a particular polysaccharide in an environment that serves as a carbon source to Bacteroidetes, thereby opening perspectives for applications in ecology, biotechnology or biomedicine. For instance, because gut microbes respond differently to specific glycans, our work has implications in the discovery and development of next generation prebiotics and synbiotics.

## Methods

### Data

The data were extracted from the PULDB database^10,16^ (http://www.cazy.org/PULDB/) in June 2018. Only fully assembled genomes deposited in NCBI Genbank or JGI IMG/M were taken. The loci were detected by the presence of *susC/D* gene pairs. The boundaries of the loci were defined by the presence of CAZyme genes within specified intergenic distances as described^10^. Information on the ecology of the various strains was retrieved from the GOLD database (https://gold.jgi.doe.gov/). Pfam assignments, taxonomical information, and the genomic position of each gene have been directly taken from the PULDB database.

### Clustering

We computed a presence/absence matrix of Glycoside Hydrolase (GH), Carbohydrate Esterase (CE) and Polysaccharide Lyase (PL) families (and subfamilies for GH5, GH13, GH30 and GH43) in each PUL, along with sulfatases (Sulf) and peptidases (Pept). Pairwise Jaccard distances^28^ between PULs have been calculated using the vegan R package. Hierarchical clustering was performed using the hclust function using the average method. The tree was at different heights using the cutree R function to define clusters with variable Jaccard distance threshold that correspond to different percentage of CAZyme composition mismatches.

### Synteny analysis

The synteny analysis has been done using the stringdist R package^46^. Proteins other than the usual PUL components (GHs, PLs, sulfatases, peptidases, transporters and regulators) have been ignored. PUL modularity has been translated as a vector of alphabetic characters to use the stringsim function of R which gives a similarity index (between 0 and 1) between two strings of characters (calculations explained in Supplementary Fig. 3). High synteny yields scores close to 1 while difference in gene order decreases this value. The synteny scores were calculated for each pair of PULs and the median of all pairwise comparisons was computed for each cluster of PULs.

### Phylogeny

The analysis was carried out with the aminoacid sequences of the SusC and SusD homologs encoded by the tandem repeat *susC/D* PULs, selecting only one representative genome for each species in our data set (Supplementary Data 12). Alignments were done using MAFFT as implemented on the GUIDANCE2 server^47^ (http://guidance.tau.ac.il/ver2/). We deleted 50% of the most uncertain positions in the resulting alignment (available upon request), and performed Maximum Likelihood analysis using FastTree on the BOOSTER server (https://booster.pasteur.fr/new/). A 1000 bootstrap replicates and branch supports Transfer Bootstrap Expectation^48^ (TBE) were computed.

### Statistics

Homogeneity in contingency tables have been tested with chi2 tests using R. Adjusted standardized residuals^49,50^ of the form:

1 $$\frac{{O - E}}{{\sqrt {\left( {E \times \left( {1 - \frac{{{\rm{RowMarginal}}}}{n}} \right) \times \left( {1 - \frac{{{\rm{ColumnMarginal}}}}{n}} \right)} \right.} }},$$

where *O, E* and *n* represent respectively the observed, expected values and the total sum of observed values.

Principal Component Analysis has been performed using FactoMineR^51^ R-package. Confidence intervals were calculated using t-test after normality verification (Shapiro test). In the case of a non-normal distribution, the confidence interval was calculated using a non-parametric Wilcoxon test. Polynomial regression was calculated used R-function lm.

### Reporting summary

Further information on research design is available in the Nature Research Reporting Summary linked to this article.

## Supplementary information

Supplementary Information

Description of Additional Supplementary Files

Supplementary Data 1

Supplementary Data 2

Supplementary Data 3

Supplementary Data 4

Supplementary Data 5

Supplementary Data 6

Supplementary Data 7

Supplementary Data 8

Supplementary Data 9

Supplementary Data 10

Supplementary Data 11

Supplementary Data 12

Reporting Summary

## Data Availability

All the raw data used in this work are provided in Supplementary Data 1. Accession numbers of the sequences used for the phylogenetic analysis of tandem repeat SusC/D proteins are given in Supplementary Data 12. A Reporting Summary for this Article is available as a Supplementary Information file. All other data supporting the findings of this study are available from the corresponding author on reasonable request.

## Code availability

Codes are provided in https://github.com/plapebie/PUL_diversity.
