# Supplementary material for: Bacteroidetes use thousands of enzyme combinations to break down glycans
Source: Nat Commun. 2019 May 3;10:2043. doi: 10.1038/s41467-019-10068-5 (PMC6499787; doi:10.1038/s41467-019-10068-5)
Supplement: Supplementary file 1 — Supplementary Information [file 41467_2019_10068_MOESM1_ESM.pdf]

Supplementary information

Bacteroidetes use thousands of enzyme combinations to break down glycans.

Lapébie *et al.*,

Supplementary Note 1 2
Supplementary Figure 1 3
Supplementary Figure 2 4
Supplementary Figure 3 5
Supplementary Figure 4 7
Supplementary Table 1 8
Supplementary Table 2 9

Supplementary Notes

Supplementary Note 1

Principal Components Analysis (PCA) of the 964 observed genomes according to the 10 following variables.

1. Number of *susC/D*-containing loci without CAZymes

2. Number of *susC/D* -containing loci without CAZymes, containing peptidases

3. Number of *susC/D* -containing loci without CAZymes, containing sulfatases

4. Number of *susC/D* -containing loci containing CAZymes (PULs)

5. Percentage of CAZyme genes outside PULs

6. Number of ORFs per PUL (median)

7. Percentage of CAZyme genes inside PULs

8. Intergenic distances outside PULs (median)

9. Intergenic distances inside PULs (median)

10. Number of genomic scaffolds

The taxonomic class is indicated as a qualitative variable in left panels. The two main dimensions explain 47.8% of the variability of the data on all 964 genomes. The projections of variables are represented by vectors and the contributions of each variable are indicated by a colour code.

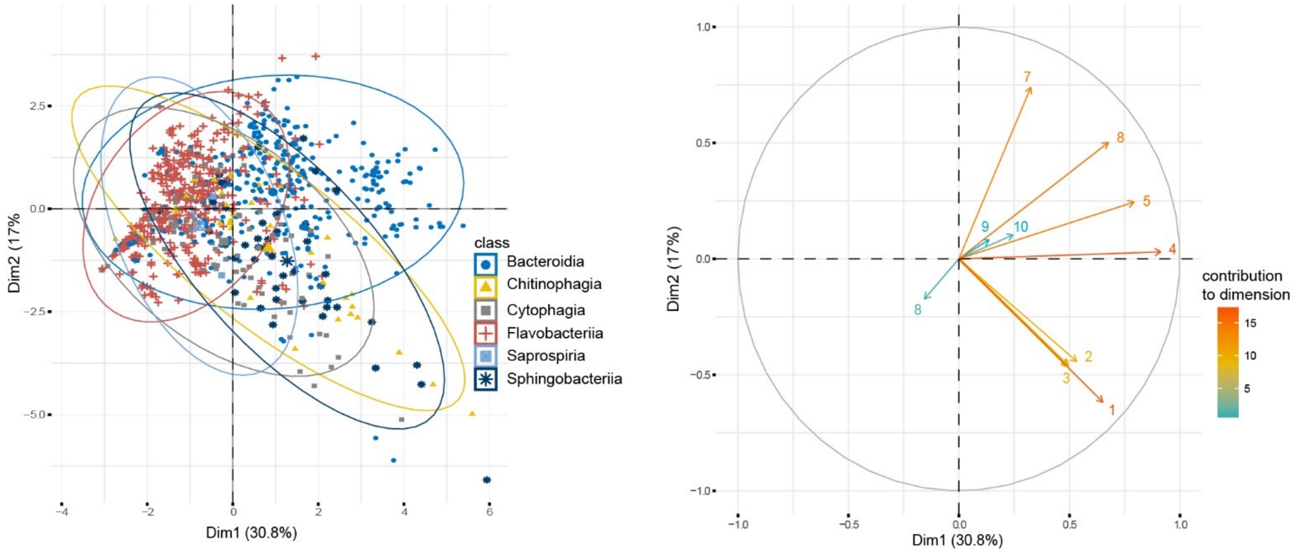

This analysis shows that:

i) The genome assembly quality (number of scaffolds) does not appear to affect significantly the number of PULs or their length.

ii) The species with the largest number of PULs are found in the Bacteroidia class.

iii) In the Chitinophaga and Shingobacteriia classes, some species harbour a large number of *susC/D* -containing loci without CAZymes, along with high proportion of CAZymes encoded outside the PULs. It is thus possible that the CAZyme genes of these species adopt a genomic organization different from that of canonical PULs.

Supplementary Figures

Supplementary Figure 1

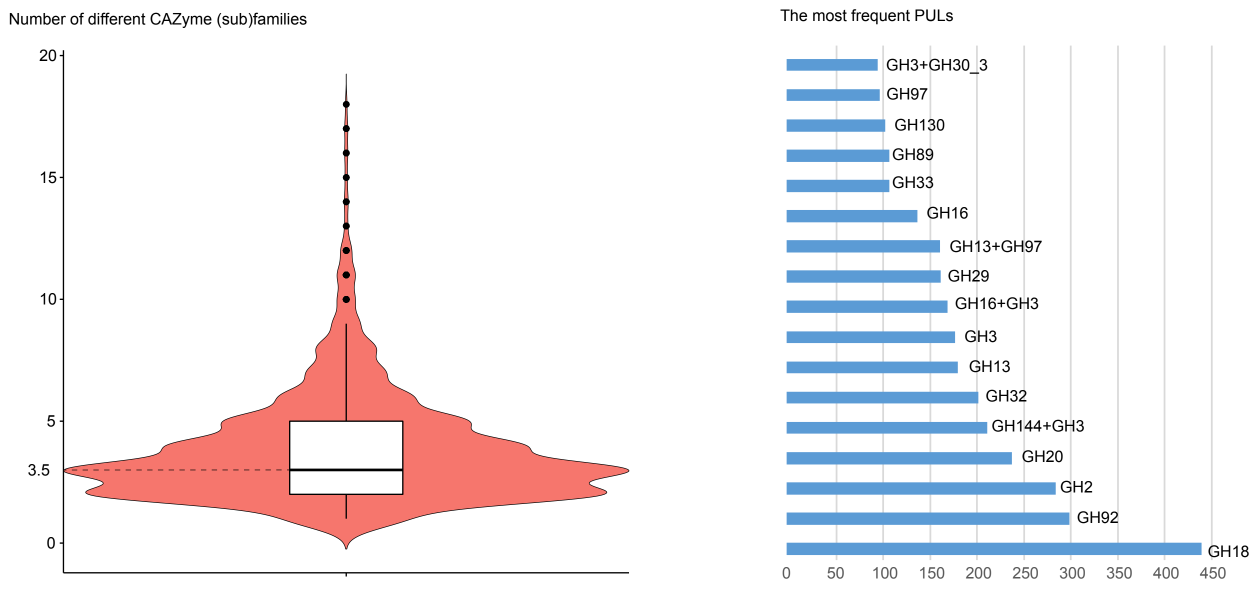

Content of the PULs. Left: Distribution of the number of different CAZyme families/subfamilies in the unique PULs with zero CAZyme mismatches. Density is represented by a coloured violinplot. In the boxplot, midline is the median, and the box represents the interquantile range (IQR) with the upper and lower limits of the box being the third and first quartile (75th and 25th percentile) respectively. Whiskers are calculated with a range of +/-1.5 IQR. Data points located outside whiskers represent outliers. Right: The most frequent PULs. The PULs encountered at least 100 times are shown along with their CAZyme composition.

Supplementary Figure 2

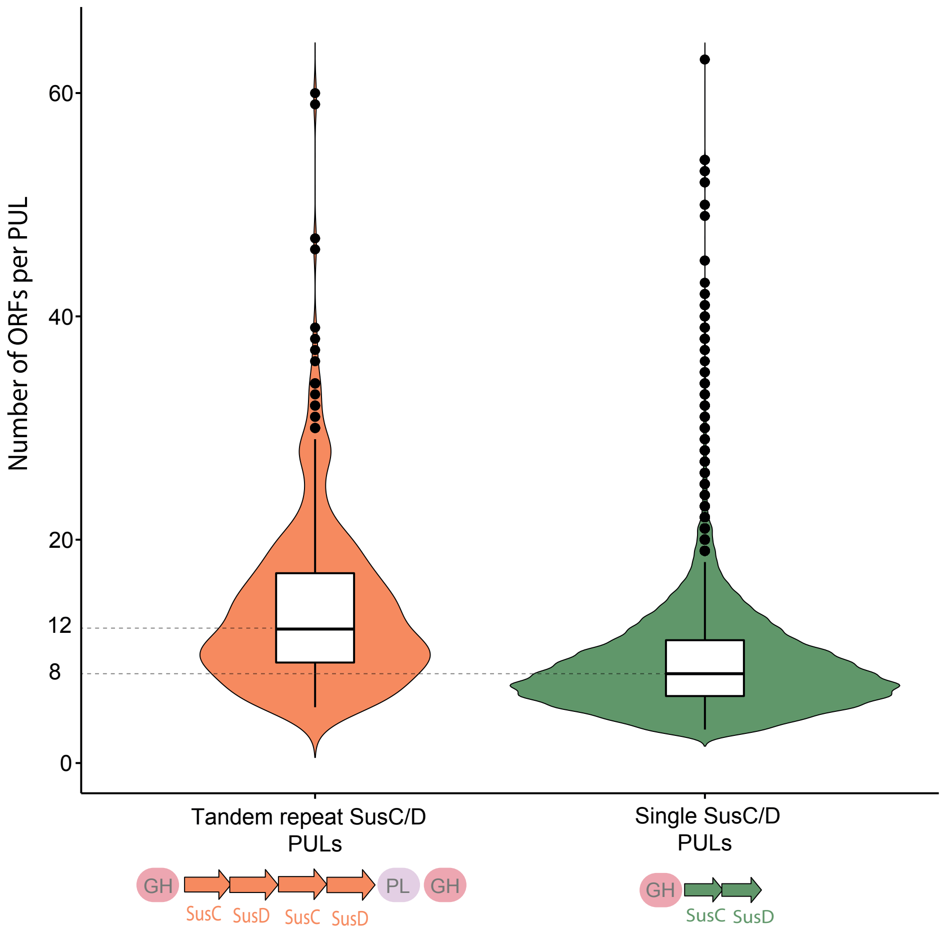

Distribution of the number of genes per PUL, distinguishing the typical canonical single *susC/D* PULs (left) and tandem repeat *susC/D* PULs (right) Density is represented by a coloured violinplot. In the boxplot, midline is the median, and the box represents the interquantile range (IQR) with the upper and lower limits of the box being the third and first quartile (75th and 25th percentile) respectively. Whiskers are calculated with a range of +/-1.5 IQR. Data points located outside whiskers represent outliers.

Supplementary Figure 3

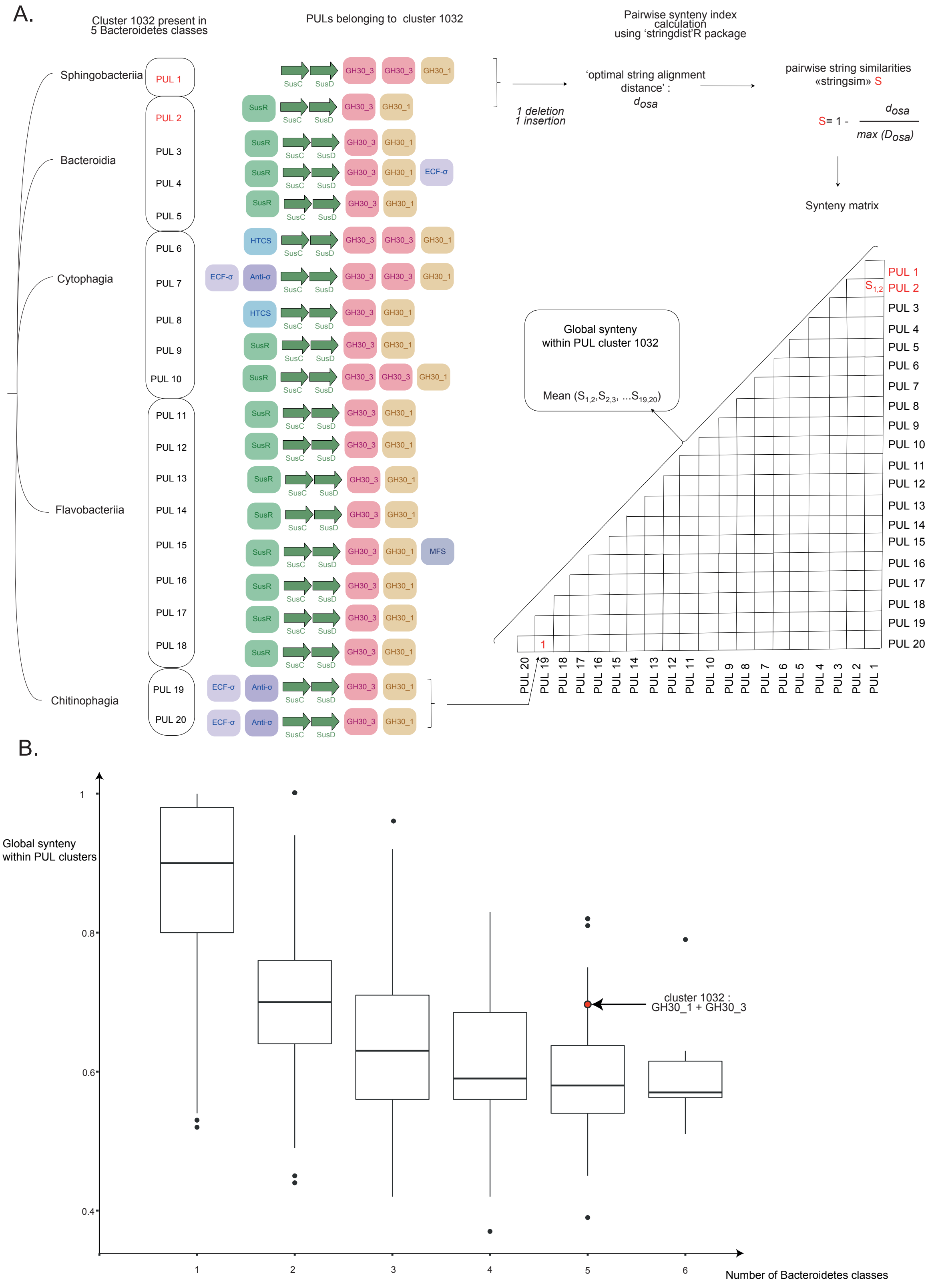

Boxplot representation of the distributions of all clusters of PULs clusters as a function of gene synteny and taxonomic distance (bottom). The vertical axis represents the median value of the synteny coefficients of each pair of PULs within a cluster. Midline is the median, and box represents the interquantile range (IQR)

with the upper lower limit of the box being the third and first quartile (75th and 25th percentile) respectively. Whiskers are calculated with a range of +/-1.5 IQR. Data points located outside whiskers represent outliers. An example of a strongly syntenic unique PUL is given in the upper part. The distributions are presented according to the number of taxonomic classes covered in the clusters.

Supplementary Figure 4

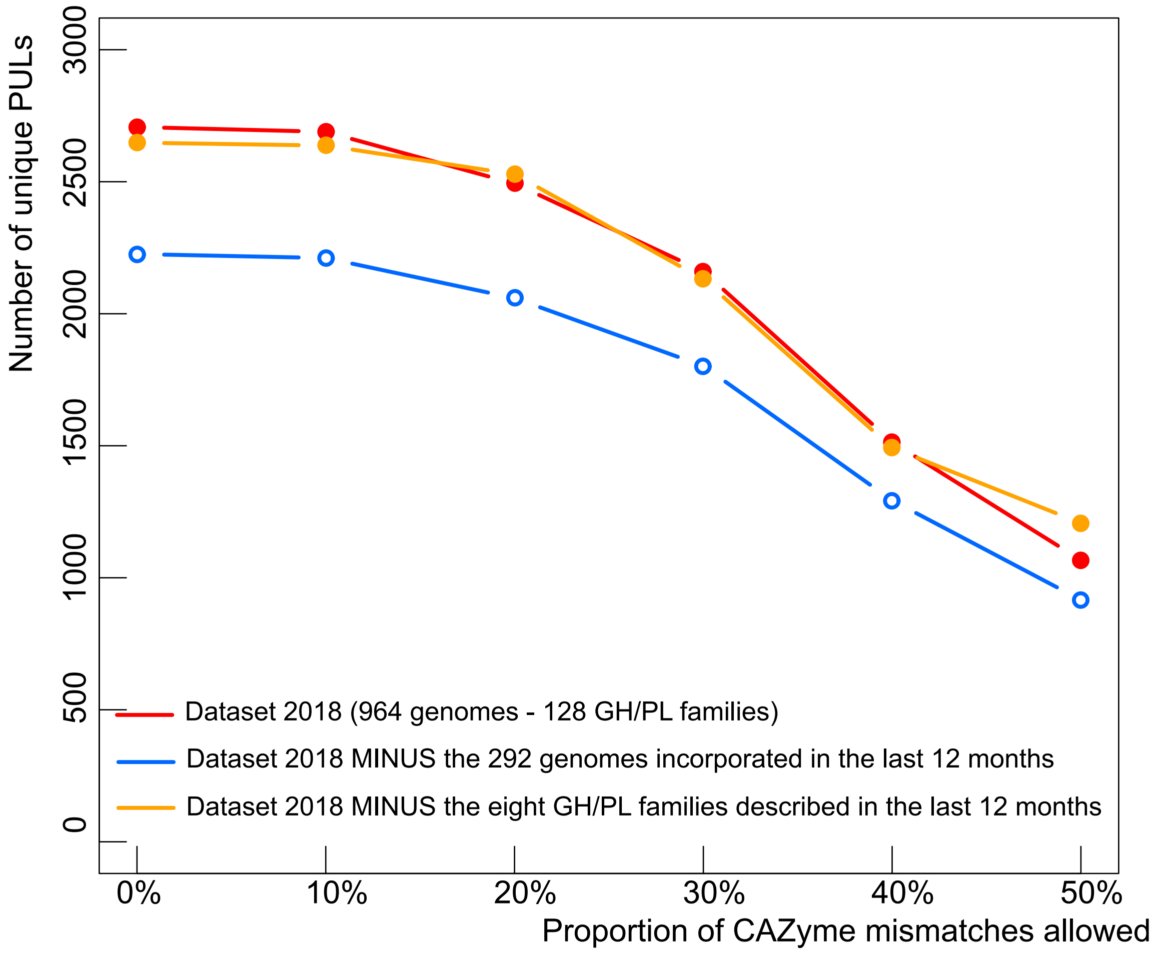

Impact of the number of families (orange) and of genomes (blue) added during the last 12 months on the number of unique PULs, with different levels of mismatches allowed during clustering.

Supplementary Tables

Supplementary Table 1

Quality of the predicted PULs against literature data. Recall indicates the percentage proportion of the gene pairs of the literature-derived PULs that we were able to predict. Precision indicates the proportion of the predicted gene pairs that are reported in the literature.

	Recall	Precision	Reference
Bacteroides ovatus ATCC 8483	0,84	0,94	1
Flavobacterium johnsoniae UW101	0,78	0,85	2
Capnocytophaga canimorsus Cc5	0,54	0,93	3
Zobellia galactanivorans DsijT	0,66	0,70	4
Bacteroides cellulosilyticus WH2 (new assembly)	0,91	0,76	5
Bacteroides thetaiotaomicron 7330 (new assembly)	0,89	0,84	6
Bacteroides ovatus ATCC 8483 (new assembly)	0,85	0,88	1

Supplementary Table 2

Tandem-repeat *susC/D* PULs and single *susC/D* PULs in the different taxonomical classes of Bacteroidetes.

Chi2- test shows significant deviation from random distribution. The standard residuals highlight the most significantly enriched classes.

	Number of loci	
**Class**	**Tandem repeat SusC/D PULs**	**Single SusC/D PULs**
Bacteroidia	424	7726
Flavobacteriia	53	1935
Sphingobacteriia	37	923
Chitinophagia	18	718
Cytophagia	34	1028
Saprospiria	1	26
	**Standard residuals (chi2 test)**	
**Class**	**Tandem repeat SusC/D PULs**	**Single SusC/D PULs**
Bacteroidia	5.91	-5.91
Flavobacteriia	-4.07	4.07
Sphingobacteriia	-0.84	0.84
Chitinophagia	-2.65	2.65
Cytophagia	-1.97	1.97
Saprospiria	-0.17	0.17
	**Pearson's Chi-squared test**	
	**X-squared = 37.805, df = 5, p-value = 4.13e-07**	
	**p-value = 4.13e-07**	

Supplementary references

1. Martens, E. C., Lowe, E. C., Chiang, H., Pudlo, N. A., Wu, M., McNulty, N. P., ... & Gordon, J. I. (2011). Recognition and degradation of plant cell wall polysaccharides by two human gut symbionts. PLoS biology, 9(12), e1001221.
2. McBride, M. J., Xie, G., Martens, E. C., Lapidus, A., Henrissat, B., Rhodes, R. G., ... & Staroscik, A. M. (2009). Novel features of the polysaccharide-digesting gliding bacterium Flavobacterium johnsoniae as revealed by genome sequence analysis. Appl. Environ. Microbiol., 75(21), 6864-6875.
3. Manfredi, P., Renzi, F., Mally, M., Sauteur, L., Schmaler, M., Moes, S., ... & Cornelis, G. R. (2011). The genome and surface proteome of Capnocytophaga canimorsus reveal a key role of glycan foraging systems in host glycoproteins deglycosylation. Molecular microbiology, 81(4), 1050-1060.
4. Barbeyron, T., Thomas, F., Barbe, V., Teeling, H., Schenowitz, C., Dossat, C., ... & Amann, R. (2016). Habitat and taxon as driving forces of carbohydrate catabolism in marine heterotrophic bacteria: example of the model algae-associated bacterium Zobellia galactanivorans DsijT. Environmental microbiology, 18(12), 4610-4627.
5. McNulty, N. P., Wu, M., Erickson, A. R., Pan, C., Erickson, B. K., Martens, E. C., ... & Gordon, J. I. (2013). Effects of diet on resource utilization by a model human gut microbiota containing Bacteroides cellulosilyticus WH2, a symbiont with an extensive glycobiome. PLoS biology, 11(8), e1001637.
6. Wu, M., McNulty, N. P., Rodionov, D. A., Khoroshkin, M. S., Griffin, N. W., Cheng, J., ... & Osterman, A. L. (2015). Genetic determinants of in vivo fitness and diet responsiveness in multiple human gut Bacteroides. Science, 350(6256), aac5992.
