# Supplementary material for: Bacteroidetes use thousands of enzyme combinations to break down glycans
Source: Nat Commun. 2019 May 3;10:2043. doi: 10.1038/s41467-019-10068-5 (PMC6499787; doi:10.1038/s41467-019-10068-5)
Supplement: Supplementary file 2 — Description of Additional Supplementary Files [file 41467_2019_10068_MOESM2_ESM.docx]

**Description of Supplementary Files**

**File Name:** Supplementary Data 1

**Description:** Raw data extracted from PULDB and analyzed here. For each locus, gene content, organism and category (Predicted PUL, Literature-derived PUL, CAZyme cluster) are given.

**File Name:** Supplementary Data 2

**Description:** List of the GH/PL enzyme families and subfamilies present in PULDB and used in this work.

**File Name:** Supplementary Data 3

**Description:** Description of each cluster of PULs (i.e. comprising at least 2 PULs) with no CAZyme mismatch. For each cluster, CAZyme composition, number of PULs and genomes, synteny coefficient are given. Information about taxonomy and known habitats is also provided. For the clusters containing literature-derived PULs, the PubMed IDs (PMIDs) of the corresponding publications are given.

**File Name:** Supplementary Data 4

**Description:** Description of each cluster of PULs (i.e. comprising at least 2 PULs) with 30% CAZyme mismatches. For each cluster, CAZyme composition, number of PULs and genomes, synteny coefficient are given. Information about taxonomy and known habitats is also provided. For the clusters containing literature-derived PULs, the PubMed IDs (PMIDs) of the corresponding publications are given.

**File Name:** Supplementary Data 5

**Description:** Families, subfamilies and their relative enrichment inside vs. outside of the PULs. Observed values and values expected according to null hypothesis are provided. Chi2 -test shows significant deviation from random distribution of CAZymes. The adjusted standardized residuals49,50 highlight the most significantly enriched CAZyme families. Degree of freedom (df) and p-values are given.

**File Name:** Supplementary Data 6

**Description:** Description of each cluster of PULs with an identical CAZyme composition and number of gene copies. For each cluster, CAZyme composition, number of PULs and genomes of origin are given. Information about taxonomy and known habitats are also provided. For the clusters containing literature-derived PULs, the PubMed IDs (PMIDs) of the corresponding publications are given.

**File Name:** Supplementary Data 7

**Description:** Number of gene copies in single family PULs organized by CAZyme families. Observed values and values expected according to null hypothesis are provided. The Chi2 -test adjusted standardized residuals49,50 shows families that are significantly more present in several copies (p-value<0.001).

**File Name:** Supplementary Data 8

**Description:** Four examples of PUL clusters exhibiting a high level of synteny across a large taxonomical breadth.

**File Name:** Supplementary Data 9

**Description:** List of proteins tagged as phosphodiesterase (Pfam: PF00149, PF01663) encoded in the vicinity of susC/D pairs by loci with no CAZymes. Proteins can be identified with their NCBI locus_tags.

**File Name:** Supplementary Data 10

**Description:** Loci lacking susC/D genes contribute weakly to the diversity of the CAZyme combinations. The composition of most of these loci is already identical to or is included in that in PULs (light and dark yellow colored lines). Original CAZyme compositions found in loci lacking susC/D genes are colored in orange.

**File Name:** Supplementary Data 11

**Description:** Number of unique PULs (0%, 10%, 20%, 30% and 40% of mismatches) after ten simulations by resampling of a growing number of genomes. 95% confidence intervals, and deviations to a normal distribution (Shapiro test) are given.

**File Name:** Supplementary Data 12

**Description:** List of the locus tags of the susC and susD genes encoding transporters of the tandem-repeat susC/D PULs. Organism name, NCBI TaxID and relative position within tandem repeat susC/D are given.
