# Supplementary material for: Bacteroidetes use thousands of enzyme combinations to break down glycans
Source: Nat Commun. 2019 May 3;10:2043. doi: 10.1038/s41467-019-10068-5 (PMC6499787; doi:10.1038/s41467-019-10068-5)
Supplement: Supplementary file 15 — Reporting Summary [file 41467_2019_10068_MOESM15_ESM.pdf]

Corresponding author(s): Bernard Henrissat

Reporting Summary

Nature Research wishes to improve the reproducibility of the work that we publish. This form provides structure for consistency and transparency in reporting. For further information on Nature Research policies, see Authors & Referees and the Editorial Policy Checklist.

Statistical parameters

When statistical analyses are reported, confirm that the following items are present in the relevant location (e.g. figure legend, table legend, main text, or Methods section).

n/a	Confirmed	
☐	☒	The exact sample size (*n*) for each experimental group/condition, given as a discrete number and unit of measurement
☒	☐	An indication of whether measurements were taken from distinct samples or whether the same sample was measured repeatedly
☐	☒	The statistical test(s) used AND whether they are one- or two-sided<br>*Only common tests should be described solely by name; describe more complex techniques in the Methods section.*
☐	☒	A description of all covariates tested
☐	☒	A description of any assumptions or corrections, such as tests of normality and adjustment for multiple comparisons
☐	☒	A full description of the statistics including central tendency (e.g. means) or other basic estimates (e.g. regression coefficient) AND variation (e.g. standard deviation) or associated estimates of uncertainty (e.g. confidence intervals)
☐	☒	For null hypothesis testing, the test statistic (e.g. *F*, *t*, *r*) with confidence intervals, effect sizes, degrees of freedom and *P* value noted<br>*Give P values as exact values whenever suitable.*
☒	☐	For Bayesian analysis, information on the choice of priors and Markov chain Monte Carlo settings
☒	☐	For hierarchical and complex designs, identification of the appropriate level for tests and full reporting of outcomes
☐	☒	Estimates of effect sizes (e.g. Cohen's *d*, Pearson's *r*), indicating how they were calculated
☐	☒	Clearly defined error bars<br>*State explicitly what error bars represent (e.g. SD, SE, CI)*

Our web collection on statistics for biologists may be useful.

Software and code

Policy information about availability of computer code

Data collection	SusC/D loci gene composition and order were taken from the PULDB database (http://www.cazy.org/PULDB/) (Terrapon et al., Nucleic Acids Res. 2018, 46(D1):D677-D683; PMID=29088389)
Data analysis	Data were analyzed using R 3.4.2 and associated packages (listed in Methods section). Amino-acid alignments and selection of the most conserved positions were obtained using the GUIDANCE2 server (http://guidance.tau.ac.il/ver2/). Phylogenetic analyses were performed using the BOOSTER server (https://booster.pasteur.fr/)

For manuscripts utilizing custom algorithms or software that are central to the research but not yet described in published literature, software must be made available to editors/reviewers upon request. We strongly encourage code deposition in a community repository (e.g. GitHub). See the Nature Research guidelines for submitting code & software for further information.

Data

Policy information about availability of data

All manuscripts must include a data availability statement. This statement should provide the following information, where applicable:

- Accession codes, unique identifiers, or web links for publicly available datasets
- A list of figures that have associated raw data
- A description of any restrictions on data availability

NCBI locus tags for each gene in all analyzed SusC/D loci are found in the PULDB database (http://www.cazy.org/PULDB/). The complete list of 13,058 PULs found in clusters of similar composition or found in singletons is available upon request. Alternatively we are happy to deposit this list as a supplementary material if the reviewers or the journal prefer.

Field-specific reporting

Please select the best fit for your research. If you are not sure, read the appropriate sections before making your selection.

☒ Life sciences ☐ Behavioural & social sciences ☐ Ecological, evolutionary & environmental sciences

For a reference copy of the document with all sections, see nature.com/authors/policies/ReportingSummary-flat.pdf

Life sciences study design

All studies must disclose on these points even when the disclosure is negative.

Sample size	Data from the PULDB database as of July 2018: 959 genomes and 30,817 SusC/D-containing loci
Data exclusions	PULDB database data from species belonging to Ignavibacteria and Gemmatimonadaceae were excluded as these taxa do not belong to Bacteroidetes. Loci harboring genes that encode glycosyltranferases were excluded as these are cytoplasmic and not periplasmic nor on the outer bacterial surface.
Replication	No wet experiment has been performed in this study. Replication is not relevant to our study.
Randomization	No wet experiment has been performed in this study. Allocation to experimental groups is not relevant to our study.
Blinding	No wet experiment has been performed in this study. Blinding is not relevant to our study.

Reporting for specific materials, systems and methods

Materials & experimental systems

n/a	Involved in the study	
☒	☐	Unique biological materials
☒	☐	Antibodies
☒	☐	Eukaryotic cell lines
☒	☐	Palaeontology
☒	☐	Animals and other organisms
☒	☐	Human research participants

Methods

n/a	Involved in the study	
☒	☐	ChIP-seq
☒	☐	Flow cytometry
☒	☐	MRI-based neuroimaging
